# Identification of necroptosis genes and characterization of immune infiltration in non-alcoholic steatohepatitis

**DOI:** 10.1186/s41065-024-00309-z

**Published:** 2024-10-01

**Authors:** Huan Zhang, Yongqiang He, Yuqing Zhao, Malina Axinbai, Yuwei Hu, Shilei Liu, Jingmin Kong, Jinhui Sun, Liping Zhang

**Affiliations:** 1 Xiyuan Hospital, China Academy of Chinese Medical Sciences, Beijing, China; 2 Beijing University of Chinese Medicine, Beijing, China; 3 Department of Digestion, Dongzhimen Hospital, Beijing University of Chinese Medicine, Beijing, China; 4 Department of Digestion, Dongfang Hospital, Beijing University of Chinese Medicine, Beijing, China; 5 Xinjiang Medical University, Urumqi, China; 6 Department of Emergency, Beijing Chaoyang Integrative Medicine Rescue And First Aid Hospital, Beijing, China

**Keywords:** Non-alcoholic steatohepatitis (NASH), Necroptosis, Bioinformatics analysis, Transcriptional factors (TFs), Immune

## Abstract

**Background:**

The most common progressive form of non-alcoholic fatty liver disease (NAFLD) is non-alcoholic steatohepatitis (NASH), which is characterized by the development of cirrhosis, and requires liver transplantation. We screened for the differentially expressed necroptosis-related genes in NASH in this study, and analyzed immune infiltration through microarray and bioinformatics analysis to identify potential biomarkers, and explore the molecular mechanisms involved in NASH.

**Methods:**

The GSE24807 microarray dataset of NASH patients and healthy controls was downloaded, and we identified the differentially expressed genes (DEGs). Necroptosis-related differential genes (NRDEGs) were extracted from these DEGs, and functionally annotated by enrichment analyses. The core genes were obtained by constructing gene co-expression networks using weighted gene co-expression network analysis (WGCNA). Finally, the transcription factor (TF) regulatory network and the mRNA-miRNA network were constructed, and the infiltrating immune cell populations were analyzed with CIBERSORT.

**Results:**

We identified six necroptosis-related genes (*CASP1*, *GLUL*, *PYCARD*, *IL33*, *SHARPIN,* and *IRF9*), and they are potential diagnostic biomarkers for NASH. In particular, *PYCARD* is a potential biomarker for NAFLD progression. Analyses of immune infiltration showed that M2 macrophages, γδ T cells, and T follicular helper cells were associated with the immune microenvironment of NASH, which is possibly regulated by *CASP1*, *IL33,* and *IRF9*.

**Conclusions:**

We identified six necroptosis-related genes in NASH, which are also potential diagnostic biomarkers. Our study provides new insights into the molecular mechanisms and immune microenvironment of NASH.

**Supplementary Information:**

The online version contains supplementary material available at 10.1186/s41065-024-00309-z.

## Introduction

The global prevalence of non-alcoholic fatty liver disease (NAFLD) is approximately 25%, paralleling the increase in the incidence of obesity, diabetes, and metabolic syndrome in recent years [1, 2]. With changes in diet and lifestyle, the incidence and mortality of NAFLD-related end-stage liver disease are expected to increase substantially [3], thereby imposing a huge burden on the patients and the healthcare system. Non-alcoholic steatohepatitis (NASH) is considered a progressive form of NAFLD [4, 5], and is characterized by liver steatosis, inflammation, and hepatocellular damage, with or without fibrosis [6]. NASH is usually diagnosed by liver biopsy at a later stage of disease progression. Given the complex pathology of NASH, there is currently a lack of non-invasive assays for the early diagnosis and monitoring of disease progression, and of effective drugs [7]. Therefore, it is crucial to explore new biomarkers for NASH and identify the genes that drive the progression of non-alcoholic fatty liver (NAFL) to NASH.

Necroptosis is a programmed cell death that is caused by various cytokines or pattern recognition receptors, and mediated by mixed lineage kinase domain-like (*MLKL*) and receptor-interacting protein kinases (*RIPKs*) [8–10]. Studies show that necroptosis not only regulates physiological processes but is also involved in ischemic and inflammatory diseases [11]. Furthermore, necroptosis is known to promote tumorigenesis and metastasis, as well as prevent tumor development when the apoptotic machinery is compromised. Therefore, necroptosis is a promising target in cancer therapy [12]. Several studies have found that necroptosis is also a common type of programmed cell death in the liver. While apoptosis is the key driver in NASH pathogenesis, necroptosis is increasingly being identified as a pathogenic factor [13]. For instance, Gautheron et al. found that the liver of NASH patients expressed high levels of RIP3 [14], and showed that RIP3-dependent necroptosis controlled liver fibrosis in a mouse model of methionine and choline-deficient diet-induced steatohepatitis [14]. Furthermore, *RIPK3*^−/−^ mice are protected against alcohol-induced liver disease [15]. Thus, necroptosis may be a promising therapeutic target for NASH, although the exact mechanisms remain to be elucidated. In addition, Furthermore, no clinical trials on the potential therapeutic effects of inhibiting necroptosis in NASH patients have been conducted so far [13].

Bioinformatics approaches are now routinely used to identify novel biomarkers for diseases from microarrays and high-throughput sequencing data [16]. To this end, we screened for necroptosis biomarkers in NASH by analyzing the GSE24807 transcriptomic dataset consisting of NASH patients and healthy controls. The necroptosis-related differentially expressed genes (NRDEGs) were identified, as well as the core genes and the regulatory transcription factors (TFs) and micro RNAs (miRNAs). Furthermore, the correlation between the NRDEGs and the infiltrating immune cell populations was also investigated. Our study provides novel insights into the molecular mechanisms of necroptosis and the immune microenvironment in NASH.

## Materials and methods

### Data collection and processing

We searched the NCBI GEO database (www.ncbi.nlm.nih.gov/geo/) for "Homo sapiens" and "NASH" as MESH terms. GSE24807, including 12 NASH samples and 5 normal samples (Table S1), was selected as the training set. In addition, GSE151158, GSE89632, GSE17470, and GSE49541 were selected as validation sets. GSE151158 included 21 normal samples and 17 NASH samples. GSE89632 included 24 normal samples and 19 NASH samples. GSE17470 included 4 normal samples and 7 NASH samples. Among the participants in GSE49541, 40 patients had mild NAFLD (fibrosis stage 0–1) whereas 32 had advanced NAFLD (fibrosis stage 3–4). Finally, 159 necroptosis-related genes (NRGs) were obtained from the Kyoto Encyclopedia of Genes and Genomes (KEGG) database (https://www.kegg.jp/entry/hsa04217; Table S2).

The platform and series matrix files of the microarray datasets were saved in text format, and annotated using R (version 4.1.3) software. Genes without corresponding gene symbols were removed during the annotation process. If a gene symbol matched more than one probe, the average of these values was used. Data integration and quantile normalization were then performed using the limma package [17] of R software.

### Identification and functional annotation of NRDEGs

The DEGs were identified using the limma package with log2 (fold change) > 1 and *p*-value < 0.05 as the criteria. The DEGs were visualized using volcano plots and heat maps using R [18]. In order to identify overlapping NRDEGs, an online tool was used to create a Venn diagram of DEGs and NRGs (http://bioinformatics.psb.ugent.be/webtools/Venn/) [19]. Gene Ontology (GO) and KEGG pathway analyses were performed on the NRDEGs using an online tool (http://www.bioinformatics.com.cn/) with *P* < 0.05 as the threshold for statistical significance [20]. The protein–protein interaction (PPI) network of the NRDEGs was constructed using STRING (http://string-db.org/), and the minimum interaction score required was set at a medium confidence level (0.4) [21].

### Weighted gene co-expression network analysis (WGCNA)

The co-expression network of the DEGs was constructed using the WGCNA package of R [22]. An appropriate soft threshold β was used to build a scale-free network. To measure gene network connectivity, the adjacency matrix was transformed into a topological overlap matrix (TOM). Genes were clustered based on the mean linkage hierarchical clustering method using the TOM dissimilarity measure. After the gene modules were determined by the dynamic shearing method, the eigenvectors of each module were calculated. The modules were then clustered, and the correlation between the modules and the disease was calculated after merging the closer modules. Finally, the DEGs most closely related to NASH were identified by drawing a Venn diagram using the VennDiagram R package [19]. The soft threshold parameter was set to β = 18 and scale-free R^2^ = 0.928.

### Immune cell infiltration

The immune cell infiltration matrix was obtained using the CIBERSORT algorithm [23]. To visualize the correlation between the 22 immune cells, the correlation heat map was performed using the "corrplot" package [24]. Correlations between hub NRDEGs and immune cells were also calculated.

### Construction of the TFs regulatory network of hub NRDEGs

The identification of putative TFs is critical to understanding the transcriptional regulation of genes. The NRDEGs-TF network was constructed using the JASPAR database (https://jaspar.genereg.net/) and then visualized using Cytoscape (version 3.8.1).

### Construction of the mRNA-miRNA regulatory network of hub NRDEGs

The miRNAs associated with the NRDEGs were predicted from six databases, including miRWalk, miRanda, microT, miRcode, miRDB, and miRmap, and target miRNAs were defined as those that were identified in at least three databases. The NRDEGs-miRNA regulatory network was visualized with Cytoscape.

### Validation of hub NRDEGs

The expression of hub NRDEGs was verified in GSE89632, GSE151158, and GSE49541. The receiver operating characteristic (ROC) curve was plotted using Hiplot (http://hiplot.com.cn). In order to assess the diagnostic specificity and sensitivity of these hub genes for NASH, the area under the curve (AUC) was calculated. Statistical significance was defined as an AUC > 0.6.

## Results

### Screening of candidate genes

**Fig. 1:**
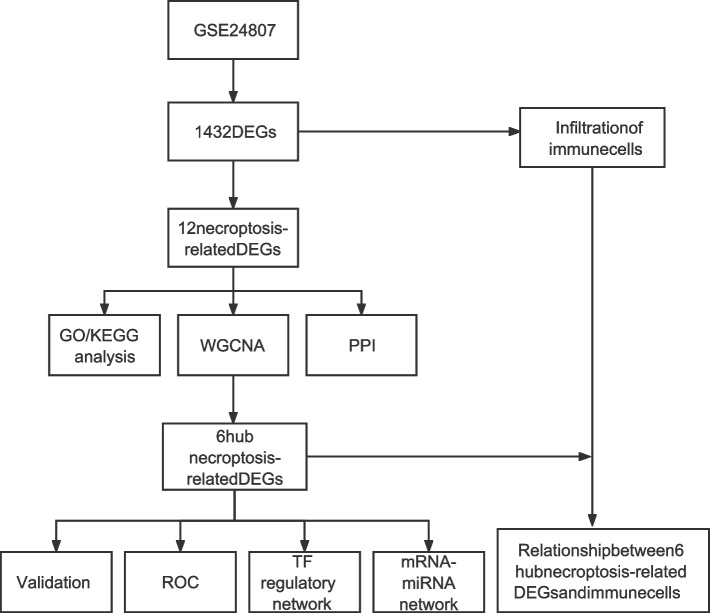
Study flow chart

**Fig. 2:**
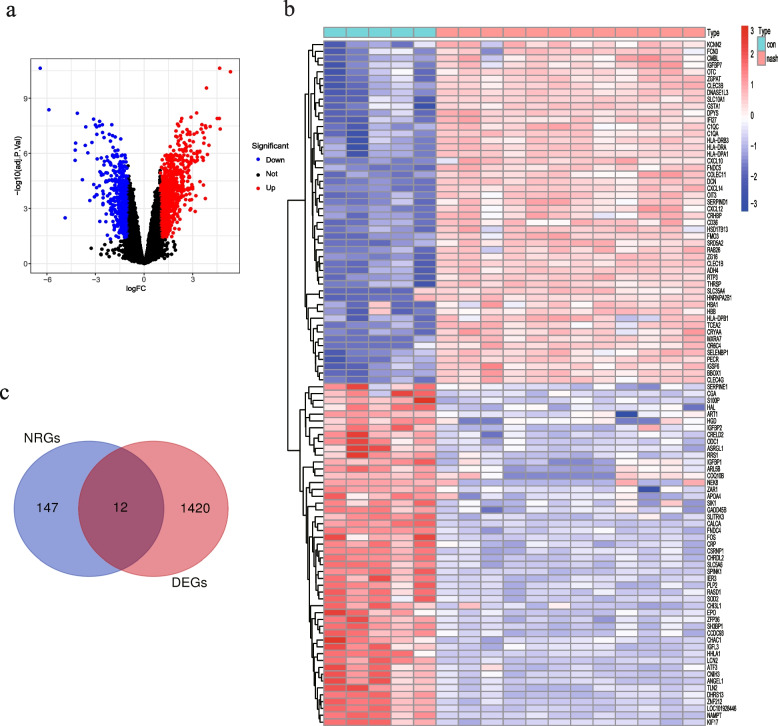
Volcano plot and Heatmap showing DEGs between NASH patients and control subjects. **a** Volcano plot. Black dots are genes not differentially expressed, red dots are genes upregulated, and blue dots are genes downregulated. **b** Heatmap of the top 100 DEGs based on the adjusted P-value and logFC. Red indicates higher expression and blue indicates lower expression. **c** The Venn diagram

The flowchart of this study is shown in Fig. 1. We identified 1432 DEGs in the liver tissues of NASH patients relative to healthy subjects, including 856 up-regulated and 576 down-regulated genes (Fig. 2a, Table S3). As shown in the heat map in Fig. 2b, the DEGs were able to separate NASH samples from control samples. Furthermore, 159 NRGs were obtained from the KEGG database, of which 12 were identified as NRDEGs based on the overlap in the Venn diagram (Fig. 2c) of DEGs and NRGs.

### Enrichment analysis and PPI network construction

**Fig. 3:**
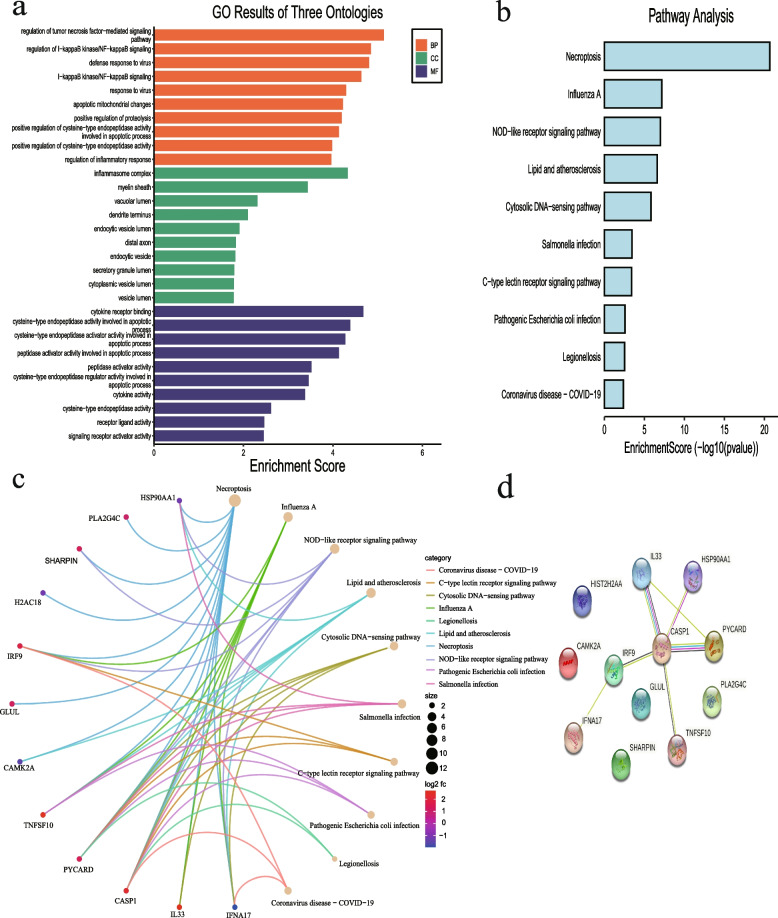
Results of the enrichment analysis and PPI network of 12 NRDEGs (**a**) GO analysis results. **b-c** KEGG analysis results. **d** PPI network

According to GO analysis, the 12 NRDEGs were enriched in biological processes (BP) such as regulation of tumor necrosis factor-mediated signaling pathway, regulation of I-kappaB kinase/NF-kappaB signaling, apoptotic mitochondrial changes, regulation of cytokine-mediated signaling pathway, response to interferon-gamma, extrinsic apoptotic signaling pathway, and positive regulation of interleukin-1 beta production. The significantly enriched cell component (CC) terms included inflammasome complex, whereas cytokine receptor binding, cytokine activity, and signaling receptor activator activity were the most significantly enriched molecular functions (MF), as shown in Fig. 3a and Table S4. Furthermore, KEGG pathway analysis revealed that the NRDEGs were mainly enriched in significantly associated with necroptosis, NOD-like receptor signaling pathway, cytokine-cytokine receptor interaction, inflammatory mediator regulation of TRP channels, lipid and atherosclerosis, glutamatergic synapse, linoleic acid metabolism, alpha-Linolenic acid, and nitrogen metabolism pathways (Fig. 3b-c, Table S5). Based on the STRING database, the PPI network of the NRDEGs was constructed, as shown in Fig. 3d.

### Results of WGCNA

**Fig. 4:**
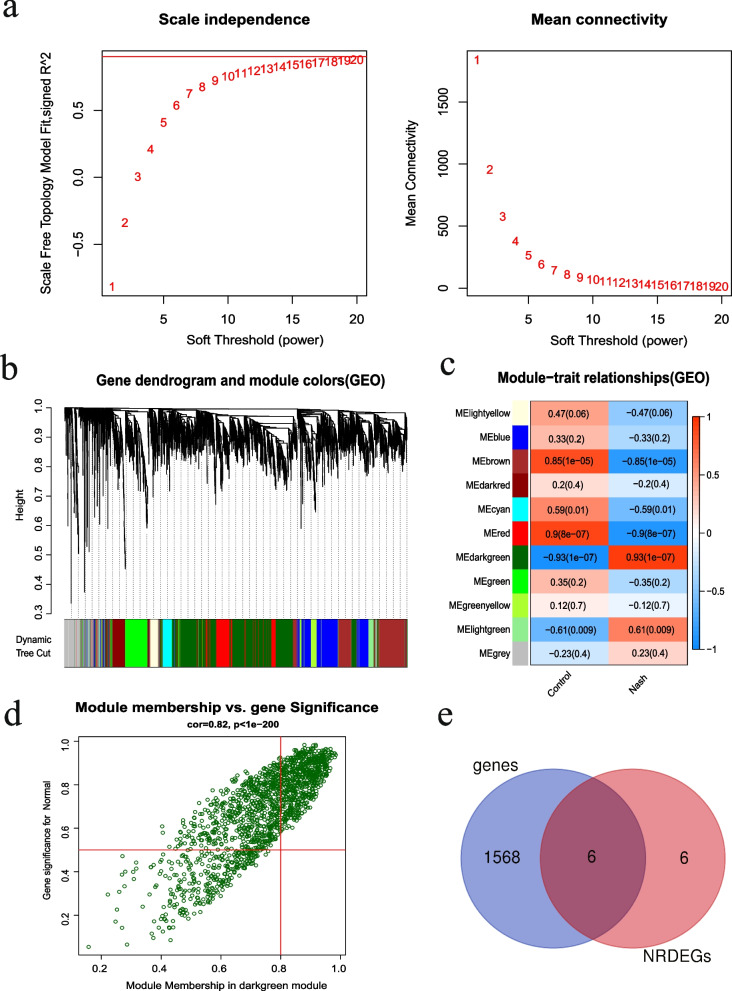
WGCNA results. **a** The soft threshold in the WGCNA algorithm. **b** Cluster dendrogram of genes. A gene module is assigned a specific color. **c** The relationship between module eigengenes and samples. The horizontal axis represents grouping. The different colors of the left vertical axis represent different modules, the correlation coefficient is indicated in each grid, and the corresponding P value is in parentheses. Darker colors indicate greater correlation, with red indicating positive correlation, and blue indicating a negative correlation. **d** Genes of the selected dark green module. **e** Venn diagram showing six hub NRDEGs

We identified NRDEGs significantly associated with NASH by WGCNA. The co-expression network was scale-free, k represented the degree of connection of the nodes, and the weighting coefficient β satisfied the condition that log (k) and log [P (k)] are negatively correlated. Based on the pickSoftThreshold function, β = 18 was selected as the appropriate soft threshold (Fig. 4a), and the corresponding scale-free topological fit index R^2^ > 0.9. The genes were clustered into 11 modules using dynamic mixed shearing (Fig. 4b-c). Based on a correlation of 0.93 and *p* < 0.001, we selected the dark green module associated with NASH as the most significant (Fig. 4d). The genes of this module and NRDEGs were intersected using Venn diagrams, and 6 hub NRDEGs were obtained (Fig. 4e), including *CASP1* (Caspase 1), *GLUL* (Glutamate-Ammonia Ligase), *PYCARD* (PYD And *CARD* Domain Containing), *IL33* (Interleukin 33), *SHARPIN* (SHANK Associated RH Domain Interactor), and *IRF9* (Interferon Regulatory Factor 9).

### Immune infiltration analysis

**Fig. 5:**
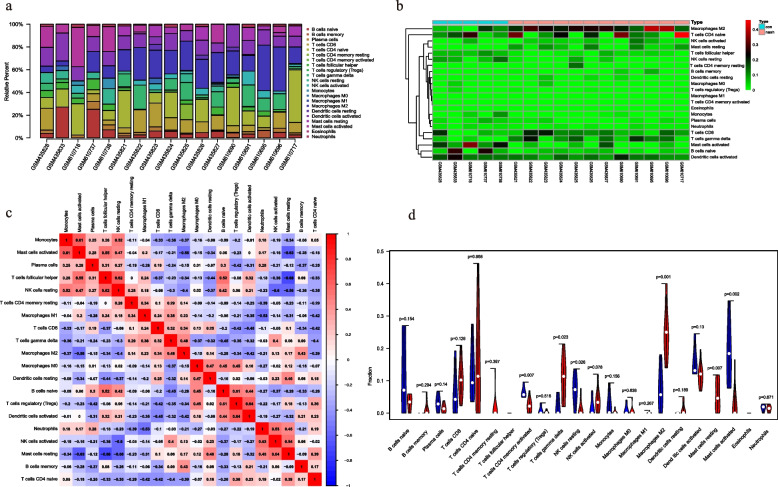
Immune infiltration analysis. **a** Sample histogram of immune cells. **b** Heatmap of the proportion of immune cells. **c** Heatmap of immune cell correlations. **d** Violin plot showing immune cell infiltration of the normal (blue) and model (red) groups

The 22 immune cells in each sample are shown in Fig. 5a and b (Table S6), and the colors represent the percentage of different immune cells. The M2 macrophages, CD4 + memory naive T cells, Mast cells activated, B cells naive, Dendritic cells activated, T cells gamma delta (γδ T cells), and CD8 + T cells were the major infiltrating immune cell types. As shown in Fig. 5c, activated mast cells and monocytes were positively correlated, as were activated dendritic cells (DCs) and regulatory T cells (Tregs). In contrast, the resting mast cells and T follicular helper (Tfh) cells were negatively correlated. Furthermore, the infiltration levels of six immune cell populations, including M2 macrophages, Mast cells activated, Mast cells resting, T cells follicular helper, T cells gamma delta, and NK cells resting, were significantly different between the two groups (*p* < 0.05; Fig. 5d).

### Relationship between hub NRDEGs and immune cells

**Fig. 6:**
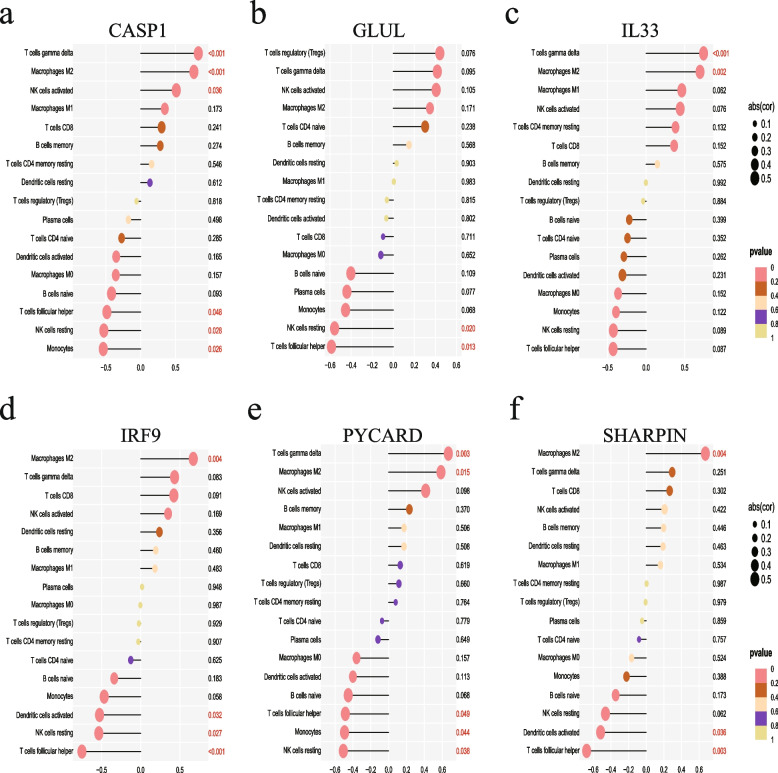
Correlation analysis of 6 hub NRDEGs with immune cell infiltration

The correlation between the major immune cells and 6 hub NRDEGs was calculated with |R|> 0.4 and *p* < 0.001 as the thresholds*.* Figure 6 shows that *CASP1* was positively correlated with M2 macrophages and the γδ T cells, and *IL33* was positively correlated with the γδ T cells. In contrast, IRF9 and Tfh cells showed a negative regulation.

### TFs regulatory network and mRNA-miRNA network

**Fig. 7:**
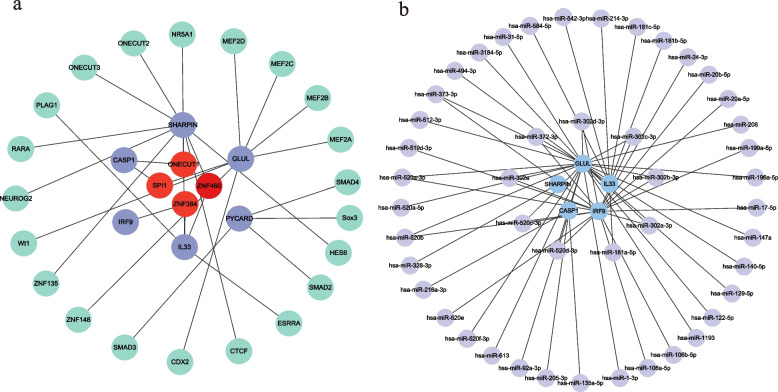
TFs Regulatory Network and mRNA-miRNA Network. **a** Gene—TFs regulatory network. Purple circles indicate hub NRDEGs, and red circles indicate TFs with the highest node degrees. **b** mRNA-miRNA Network. Blue circles indicate hub NRDEGs

We obtained 30 gene-TFs pairs for the 6 hub NRDEGs (Table S7) and constructed a gene-TFs regulatory network consisting of 31 nodes and 29 edges (Fig. 7a). *ONECUT1*, *SPI1*, *ZNF460*, and *ZNF43* had the highest node degrees. In addition, we obtained 47 targeted miRNAs of 5 hub NRDEGs and identified 58 mRNA-miRNA pairs, which were not predicted by *PYCARD* under the screening conditions (Fig. 7b, Table S8).

### Validation and diagnostic value of the hub NRDEGs

**Fig. 8:**
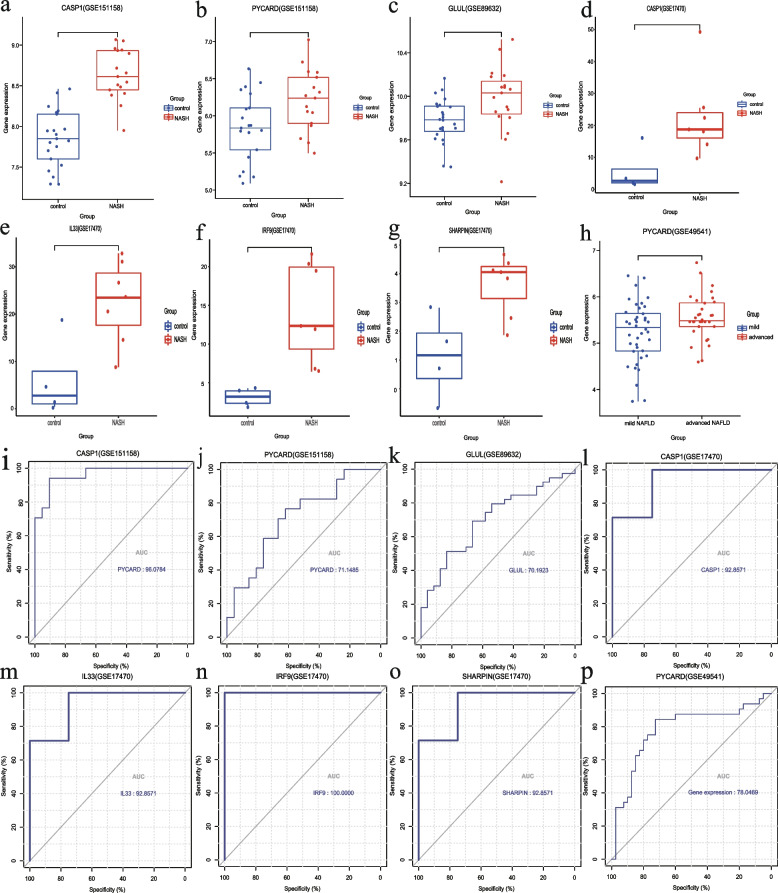
Validation of hub genes. **a-g** Detailed expression of six hub NRDEGs in NASH samples and healthy samples. **h** Detailed expression of *PYCARD* in mild NAFLD and advanced NAFLD. **i–o** Diagnostic performance of six hub NRDEGs in NASH samples and healthy samples. **p** Diagnostic performance of *PYCARD* in mild NAFLD and advanced NAFLD

The expression of six hub NRDEGs was validated in the GSE151158, GSE89632, and GSE17470 datasets, and that of the six hub NRDEGs was consistent with predicted results (Fig. 8a-g). According to the GSE49541 dataset, the expression of *PYCARD* was also significantly higher in the advanced NAFLD group (Fig. 8h). ROC curves were plotted to evaluate the sensitivity and specificity of six hub NRDEGs in NASH diagnosis, which indicated that all of these were diagnostically relevant (Fig. 8i-o). Furthermore, CASP1, IL33, IRF9,and SHRAPIN showed high diagnostic accuracy, whereas *PYCARD* was also identified as a biomarker for NAFLD progression (Fig. 8p).

## Discussion

The global prevalence of NAFLD is nearly 25% [25], which imposes a considerable socio-economic burden. NASH is an inflammatory subtype of NAFLD [5], and its steadily increasing incidence rate warrants novel treatment strategies. Therefore, it is imperative to explore its molecular mechanisms and identify new biomarkers. Recent studies have shown that necroptosis is a key pathological driver of NASH, and a potential source of novel diagnostic markers and therapeutic targets.

Our study aimed to identify biomarkers associated with necroptosis in NASH and explore the association between necroptosis and immune cell infiltration. We retrieved 159 NRGs from the KEGG database, of which 12 were differentially expressed between the NASH samples and controls in the GSE24807 dataset. GO analysis further indicated that these NRDEGs were enriched in the regulation of cytokine-mediated signaling pathways, the regulation of I-κB kinase/NF-κB signaling, the response of interferon γ, positive regulation of cysteine-type endopeptidase activity, and the regulation of mitochondrial changes during apoptosis. Likewise, the significantly enriched KEGG pathways included those of necroptosis, JAK-STAT signaling pathway, NOD-like receptor signaling pathway, inflammatory mediator-regulated signaling via TRP channel, lipid and atherosclerosis, alpha-linolenic and linoleic acid metabolism, and nitrogen metabolism.

NASH-related modules were constructed by WGCNA, and six hub NRDEGs closely related to NASH were finally identified, of which most have not been reported previously in the context of NASH development. Furthermore, ROC analysis established that *CASP1*, *GLUL,* and *PYCARD* have diagnostic significance for NASH, and PYCARD can also serve as a diagnostic marker for NAFLD progression.

*CASP1*, an interleukin-1β converting enzyme (ICE), showed the highest diagnostic accuracy for NASH. It plays a key role in apoptosis, pyroptosis, inflammatory response, and innate immunity, and is a key enzyme in the apoptotic pathway [26, 27]. *CASP1* has been reported to induce apoptosis in animal models of ischemic brain injury, and familial amyotrophic lateral sclerosis [28, 29]. Furthermore, *CASP1* and its activator NLRP3 are the core components of the inflammasome complex [30], and the cleavage of *CASP1* by Gasdermin D is the trigger of pyroptosis [31]. Recent studies have shown that caspase 8 is a molecular switch of necroptosis during late embryonic development, and *CASP1* lies downstream of activated caspase 8 [32]. There are several substrate proteins involved in *CASP1* splicing and processing, and their functions are highly complex. Based on current evidence, the mechanisms underlying the pathophysiological role of *CASP1* in NASH are unclear.

We found that the liver tissues of NASH patients expressed significantly higher levels of *GLUL* compared to those of healthy controls. *GLUL* is the only known glutamine synthase that catalyzes the conversion of ATP from glutamate and ammonia to glutamine, and is involved in ammonia and glutamate detoxification, cell signaling, cell proliferation, and acid/base homeostasis [33]. The glutamine-glutamate ratio is associated with blood pressure, triglycerides, and glucose levels [34]. Petrus et al. compared metabolites produced by white adipose tissue in 81 obese and non-obese women, and found that *GLUL* was the most significantly dysregulated gene in the glutamine pathway in obese patients [35]. Recent studies have shown that *GLUL* is involved in RIP3-dependent necroptosis [36], although its pathological role in NASH remains to be elucidated.

*PYCARD*, also known as ASC (Apoptosis-associated speck-like protein containing a caspase recruitment domain), is a pro-apoptotic protein [37] and an adaptor molecule of the inflammasome complex that activates caspase-1, and promotes the secretion of inflammatory cytokines [38]. Extracellular *PYCARD* may induce autoantibody production, thereby regulating innate and adaptive immune responses [39]. Thus, circulating *PYCARD* is a serum biomarker of inflammation and autoimmune diseases such as systemic lupus erythematosus and rheumatoid arthritis [40]. Fritsch et al. found that catalytically inactive CASP8 induced ASC formation [41]. In our study, *PYCARD* was overexpressed in NASH samples and regarded as a biomarker for NAFLD progression. However, the specific pathological mechanisms need to be investigated further.

*IL-33* is a pro-inflammatory cytokine of the *IL-1* superfamily and plays a vital role in inflammation, and cancer, and central nervous system diseases [42]. Recent studies have shown that necroptosis directly induces *IL-33* release, which activates basophils and eosinophils [43]. In addition, It has been found that patients with liver cirrhosis have an increased level of IL-33 [44]. However, another study showed that *IL-33* deficiency did not affect the severity of liver inflammation or liver fibrosis in a mouse model of diet-induced steatohepatitis [45]. Thus, the exact role of *IL-33* in the liver needs to be elucidated further.

As part of the linear ubiquitin chain assembly complex (LUBAC), *SHARPIN* regulates protein ubiquitination and signal transduction [46]. Monoubiquitination regulates immune signaling and cell death (including apoptosis and necroptosis) [47, 48]. Sieber et al. observed extensive liver injury and premature death in *SHARPIN*-deficient mice [49]. IRF9 regulates interferon-driven gene expression, and alleviates hepatic insulin resistance, steatosis and inflammation through interaction with PPARα [50]. However, McComb et al. showed that macrophages with IRF-9-STAT1/STAT2 deficiency are highly resistant to necroptosis [51]. The relationship between the protective effect of IRF9 and necroptosis in NASH needs further investigation.

Several studies have shown that the local immune microenvironment greatly contributes to NASH development and progression [52]. To this end, we used the CIBERSORT algorithm to analyze the infiltrating immune cell populations in NASH, and found that the M2 macrophages and γδ T cells were elevated in NASH liver tissues and were the predominant infiltrating cells. A previous study had shown that NASH accelerated HCC progression by promoting M2 macrophage polarization via upregulation of IL-10 [53]. In addition, the γδ T cells contribute to the development and progression of autoimmune liver disease [54]. We next analyzed the correlation between hub NRDEGs and the infiltrating immune cell types, and found that CASP1 was positively correlated with M2 macrophages and γδ T cells, and IL-33 was positively correlated with the γδ T cells. Thus, CASP1 and IL33 may contribute to the progression of NASH by modulating the local immune responses. In addition, the proportion of Tfh cells was lower in the diseased liver compared to the normal liver tissues, and correlated negatively with IRF9 expression. There is evidence that Tfh cells contribute to the progression of atherosclerosis [55], and may play a key role in regulating adipose tissue inflammation in obesity-induced type 2 diabetes [56]. Therefore, we speculate that IRF9 may exert a protective effect against NASH by inhibiting the Tfh cells, which will have to be validated further.

We established the networks of hub NRDEGs with TFs and miRNAs to further assess their role in NASH occurrence and development at the transcriptome level. We identified 4 TFs that closely interact with hub NRDEGs, namely ONECUT1, SPI1, ZNF460, and ZNF43. ONECUT1, a transcription factor belonging to the cut homeobox family, is mainly enriched in the liver and regulates the cell cycle and glucose metabolism [57]. The sub-network of ONECUT1 consists of 44 differentially expressed genes, many of which are involved in fatty acid metabolism and are highly correlated with the progression of steatosis [58]. SPI1 is primarily expressed in bone marrow cells and lymphocytes [59], and correlates positively with insulin resistance and inflammation in NASH patients, making it a potential therapeutic target [60]. ZNF460 and ZNF43 are members of the zinc finger protein family, which play an essential role in regulating cell proliferation, differentiation, and metabolism [61]. The roles of ZNF460 and ZNF43 in NASH have not been elucidated so far.

We also obtained 58 mRNA-miRNA pairs, and hsa-miR-372-3p, hsa-miR-520a-3p, hsa-miR-520b, hsa-miR-520c-3p, hsa-miR-520d-3p, hsa-miR-302a-3p, hsa-miR-302b-3p, hsa-miR-302c-3p, hsa-miR-302d-3p, and hsa-miR-302e in particular were closely related to the NRDEGs. Has-miR-372-3p is involved in lipid metabolism, and rapamycin causes triglyceride accumulation by downregulating the expression of has-miR-372-3p [62]. Low expression levels of has-miR-372–3p have been associated with a poor prognosis of HCC [63]. Therefore, the potential roles of the GLUL-has-miR-372-3p and IRF9-has-miR-372-3p regulatory networks in NASH deserve further investigation. The has-miR-302 family is involved in cell differentiation, proliferation and immune responses, and acts as tumor suppressor genes in most tumors. Additionally, miR-302a is known to promote chronic inflammatory responses in atherosclerosis [64]. Therefore, hsa-miR-302 and has-miR-372-3p are promising therapeutic targets in NASH.

Although we were able to identify some novel biomarkers of NASH related to necroptosis and immune cell infiltration, there are some limitations of this study that ought to be considered. First, the study does not provide clinically relevant information, such as drug use. Second, there is currently no systematic database of necroptosis-related genes, and more genes remain to be discovered. Finally, the results of this study have not been validated through in vivo and in vitro experiments, which needs to be addressed in future studies.

## Conclusion

We identified 6 necroptosis-related hub genes in NASH, namely *CASP1*, *GLUL*, *PYCARD*, *IL33*, *SHARPIN,* and *IRF9*, and they can diagnose NASH reasonably. In addition, *PYCARD* was also identified as a diagnostic marker for NAFLD progression. Furthermore, an increase in M2 macrophages and γδ T cells, and a decrease in Tfh cells may be associated with NASH pathogenesis, and correlated with CASP1, IL33, and IRF9. Therefore, our study provides new insights into molecular mechanisms of NASH, along with potential diagnostic biomarkers.

## Supplementary Information

**Additional file 1: Table S1.** Clinical information for the datasets.

**Additional file 2: Table S2.** 159 necroptosis-related genes

**Additional file 3: Table S3.** DEGs in the database.

**Additional file 4: Table S4.** Go analysis results.

**Additional file 5: Table S5.** KEGG pathway results.

**Additional file 6: Table S6.** Resule of inmuune cell infiltration.

**Additional file 7: Table S7.** The potential TFs of 6 hub NRDEGs

**Additional file 8: Table S8.** miRNAs interact with mRNAs.

## Data Availability

The following information on data availability is provided: Raw measurements are provided in the Supplementary file. Further inquiries can be directed to the corresponding authors.

## Abbreviations

GEO: Gene Expression Omnibus
DEGs: Differentially expressed genes
NRDEGs: Necroptosis-related differential genes
NRGs: Necroptosis-related genes
GO: Gene Ontology
KEGG: Kyoto Encyclopedia of Genes and Genomes
PPI: Protein-protein interaction
WGCNA: Weighted gene co-expression network analysis
TFs: Transcription factors
NASH: Non-alcoholic steatohepatitis
NAFLD: Non-alcoholic fatty liver disease
NAFL: Non-alcoholic fatty liver
*MLKL*: Mixed lineage kinase domain-like
*RIPKs*: Receptor-interacting protein kinases
TOM: Topological overlap matrix
ROC: Receiver operating characteristic
AUC: Area under the curve
BP: Biological processes
CC: Cell component
MF: Molecular functions
CASP1: Caspase 1
GLUL: Glutamate-Ammonia Ligase
PYCARD: PYD and CARD Domain Containing
IL33: Interleukin 33
SHARPIN: SHANK Associated RH Domain Interactor
IRF9: Interferon Regulatory Factor 9
ICE: Interleukin-1β converting enzyme
LUBAC: Linear ubiquitin chain assembly complex
