# Supplementary material for: Identification of necroptosis genes and characterization of immune infiltration in non-alcoholic steatohepatitis
Source: Hereditas. 2024 Oct 1;161:32. doi: 10.1186/s41065-024-00309-z (PMC11443769; doi:10.1186/s41065-024-00309-z)
Supplement: Supplementary file 7 — Additional file 7: Table S7. The potential TFs of 6 hub NRDEGs [file 41065_2024_309_MOESM7_ESM.docx]

**The potential TFs of 6 hub NRDEGs**

| **Genes** | **TFs** | | | |  | **Count** | |
| --- | --- | --- | --- | --- | --- | --- | --- |
| **CASP1** | |  | **SPIB, SPI1, ONECUT1** | | | **3** |  |
| **GLUL** | |  | **SPI1, Wt1, ZNF148, MEF2A, MEF2B, MEF2C, MEF2D, CDX2** | | | **8** |  |
| **IL33** | |  | **ZNF460, ZNF384, PLAG1, ESRRA, NR5A1** | | | **7** |  |
| **IRF9** | |  | **ZNF384** | | | **6** |  |
| **PYCARD** | |  | **SMAD2, SMAD3, SMAD4, Sox3** | | | **4** |  |
| **SHARPIN** | |  | **ONECUT1, ONECUT2, ONECUT3, NEUROG2, HES6, CTCF, RARA, NF460, ZNF135** | | | **8** |  |
|  | |  | |  | |  | |
|  | |  | |  | |  | |
|  | |  | |  | |  | |
|  | |  | |  | |  | |
|  | |  | |  | |  | |
|  | |  | |  | |  | |
|  | |  | |  | |  | |
|  | |  | |  | |  | |
|  | |  | |  | |  | |
|  | |  | |  | |  | |
|  | |  | |  | |  | |
|  | |  | |  | |  | |
|  | |  | |  | |  | |
|  | |  | |  | |  | |
|  | |  | |  | |  | |
|  | |  | |  | |  | |
|  | |  | |  | |  | |
|  | |  | |  | |  | |
|  | |  | |  | |  | |
|  | |  | |  | |  | |
|  | |  | |  | |  | |
|  | |  | |  | |  | |
|  | |  | |  | |  | |
|  | |  | |  | |  | |
|  | |  | |  | |  | |
|  | |  | |  | |  | |
